# A Quadrotor UAV Aeromagnetic Compensation Method Based on Time–Frequency Joint Representation Neural Network and Its Application in Mineral Exploration

**DOI:** 10.3390/s25185774

**Published:** 2025-09-16

**Authors:** Ping Yu, Guanlin Huang, Jian Jiao, Longran Zhou, Yuzhuo Zhao, Pengyu Lu, Lu Li, Shuiyan Shi

**Affiliations:** Department of Solid Earth Geophysics, College of Geo-Exploration Science and Technology, Chaoyang Campus, Jilin University, Changchun 130012, China; yuping@jlu.edu.cn (P.Y.); glhuang23@mails.jlu.edu.cn (G.H.);

**Keywords:** quadcopter UAV, aeromagnetic compensation, continuous wavelet transform, Bi-LSTM, time-frequency joint

## Abstract

Quadrotor UAV-based aeromagnetic survey for mineral exploration has become a crucial solution in modern airborne geophysics due to its prominent advantages of cost-effectiveness and high efficiency. During the detection process, the magnetic anomaly interference generated by the quadrotor UAV itself reduces the signal-to-noise ratio (SNR) of the target signal, and some noise overlaps with the target signal in both time and frequency domains. Traditional methods exhibit poor compensation capability for such noise. To address these issues, this paper proposes an aeromagnetic compensation method based on a time–frequency joint representation neural network. This method combines continuous wavelet transform (CWT) and bidirectional long short-term memory (Bi-LSTM) to establish a prediction model. It uses wavelet transform to extract the frequency variation characteristics of the UAV’s magnetic interference, and it inputs these frequency characteristics along with the original time-domain data into the Bi-LSTM network to predict the UAV’s noise. Bi-LSTM can effectively extract the temporal logical connections in time-series signals, thereby improving the accuracy of the compensation model and ensuring high robustness. In this study, magnetic interference data from quadrotor UAV compensation flights were collected for experiments to evaluate the performance of the proposed method. Experimental results show that the neural network fused with time–frequency features, when applied to UAV aeromagnetic compensation, significantly enhances the accuracy and robustness of the compensation method. To verify the method’s effectiveness in removing UAV-generated noise during actual exploration, aeromagnetic survey data from a specific area were compensated using this method.

## 1. Introduction

UAV aeromagnetic exploration has been widely used in geological surveys, mineral exploration, and other fields due to its technical advantages such as low cost and wide application range [1,2,3,4]. Currently, quadrotor UAVs are extensively employed in aeromagnetic exploration. During aeromagnetic survey operations, magnetometers receive not only the target magnetic field signals but also various noise interferences, including the Earth’s magnetic field, diurnal magnetic field variations, UAV-generated magnetic interference, and geomagnetic gradients. These interferences need to be separated and eliminated through effective technical means. Most magnetic interferences can be detected using independent detectors; however, the magnetic interference generated by the UAV itself cannot be directly detected by independent detectors. Therefore, it is necessary to indirectly predict and remove the UAV-induced magnetic interference using detector data, a process known as aeromagnetic compensation.

To quantify the UAV-generated magnetic interference, Tolles and Lawson first analyzed the mechanism of magnetic interference from flight platforms [5]. They classified the magnetic interference into three categories based on its sources: constant magnetic field, induced magnetic field, and eddy current magnetic field, and they established a mathematical model of magnetic interference related to maneuvering actions, which is known as the Tolles–Lawson equation (abbreviated as the T-L equation hereinafter). Based on the T-L equation, Leliak designed an aeromagnetic compensation flight scheme and a method to estimate magnetic interference compensation during maneuvering [6]. The essence of this method is to solve the compensation coefficients in an overdetermined system of equations using data obtained from compensation flights. Consequently, to improve the quality of the solved compensation coefficients, the calculation methods have been continuously optimized. In 1979, Bickel proposed a small-signal compensation method [7], which can effectively calculate approximate compensation coefficients. In 1990, Leach improved the least squares method [8] and proposed using ridge regression to solve the T-L equation. In 2008, Praga developed an improved ridge regression algorithm to enhance calculation accuracy [9]. In 2010, Zhang et al. combined Principal Component Analysis (PCA) with the least squares method [10] and proposed the partial least squares method. Dou proposed a real-time compensation method based on Recursive Least Squares (RLS) [11], enabling real-time calculation.

The aforementioned methods are based on traditional linear regression and require consideration of the linear correlation between data, leading to issues such as algorithm instability and poor robustness. In 1993, Williams proposed the application of neural networks in aeromagnetic compensation [12], establishing a neural network model for flight platforms. Attitude information and time information were used as inputs, and aeromagnetic interference was used as output to train the model for estimating aeromagnetic interference. In 2017, Ma evaluated the performance of neural networks [13] and introduced observation noise to suppress the overfitting problem of neural networks. The accuracy of traditional compensation methods [14,15,16,17,18] is gradually unable to meet the increasingly higher accuracy requirements of aeromagnetic exploration. In 2021, Zhao proposed the Denoising Autoencoder (DAE) method [19], which improves the speed and accuracy of aeromagnetic compensation through deep autoencoding. In the same year, Zhao applied the Generalized Regression Neural Network (GRNN) to aeromagnetic compensation [20]. In 2024, Lou proved through simulations and experiments that Bi-LSTM can effectively extract time-domain information, capture data correlations between time series, and better identify magnetic anomalies [21]. Many studies [22,23,24] have also proven that neural networks achieve better compensation effects than traditional compensation methods in addressing aeromagnetic compensation problems. Previous compensation methods focused on analyzing the time-domain information of UAV noise and separated filtering and compensation into two separate steps, which reduced compensation accuracy and compromised the stability of the compensation method.

The signal-to-noise ratio (SNR) of signals obtained from actual aeromagnetic exploration is relatively low. Fourier transform can simply divide noise signals into high-frequency and low-frequency components. In 2004, Gamey analyzed the frequency of magnetic interference generated by the rotor blades of rotary-wing helicopters [25] and found that the signal frequency is related to the number of rotors. In 2010, Dou designed an adaptive wavelet filter [26], which retains the magnetic interference of the flight platform while removing other interferences through filtering. In 2020, Walter further analyzed the sources of signals received by optically pumped magnetometers and their corresponding frequency ranges [27], facilitating the extraction of required signals from the frequency domain. Although the aforementioned methods can remove noise with distinct high-frequency characteristics through filtering, the frequency of UAV-generated magnetic interference is close to that of magnetic anomalies generated by detected targets. Thus, UAV-generated magnetic interference cannot be simply removed by filtering. In this paper, continuous wavelet transform is applied to the time-domain signals of UAV noise to obtain frequency characteristics. By comparing and analyzing the time–frequency spectra of three compensation flight experiments with the same maneuvering actions but different altitudes, it is verified that there is a strong correlation between UAV noise and flight attitude.

Previous aeromagnetic compensation methods, which only use time-domain information, achieve good compensation results for single-rotor and fixed-wing aircraft but poor results for quadrotor UAVs. Since most quadrotor UAVs adopt a demagnetized structure, changes in flight attitude depend on the rotation frequency adjustment of motors on each rotor [28]. Therefore, the variation of magnetic interference in quadrotor UAVs is strongly correlated with the rotation frequency of the motors. Consequently, aeromagnetic compensation for quadrotor UAVs needs to consider frequency variation characteristics. This paper proposes a time–frequency joint representation aeromagnetic compensation method (Bi-FT). Time-domain information is used to analyze the UAV’s attitude, and frequency information is used to analyze changes in motor speed. A bidirectional long short-term memory network is employed to predict the magnetic interference characteristics generated by the quadrotor UAV during specific maneuvers.

The aeromagnetic compensation method proposed in this paper was tested on actual exploration data. The study area is located in Dandong, southeastern Liaoning Province, China, which is situated on the northeastern margin of the North China Craton and the core part of the Jiaoliao Block, serving as an important component of the North China Craton. It has a long geological history, with crustal evolution exceeding 3.8 billion years. As a craton margin, Dandong has been affected by multiple tectonic events, particularly during the Mesozoic, when continuous subduction of the Pacific Plate beneath the Eurasian Plate triggered multi-stage deformation and magmatic activity in the region. In terms of geological structure, Dandong is located in the hanging wall of the north-dipping Dabie–Sulu suture zone. This special location led to complex deformation and magmatic events during the Mesozoic, driven by the subduction of the Paleo–Pacific Plate toward the Eurasian Plate. Among them, the NE-trending Jixingou Fault Zone is a major structure in the region, which has induced moderate to strong seismic activity in history and contains a large number of different ore veins. It serves as a key window for studying the tectonic evolution of the East Asian continental block.

**Figure 1:**
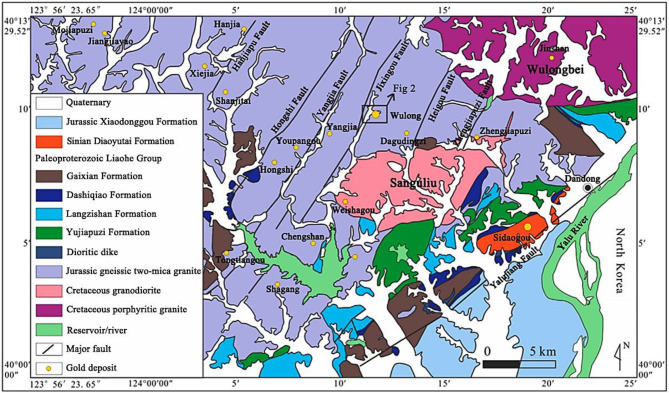
Regional geological map of Wulong gold deposit.

**Figure 2:**
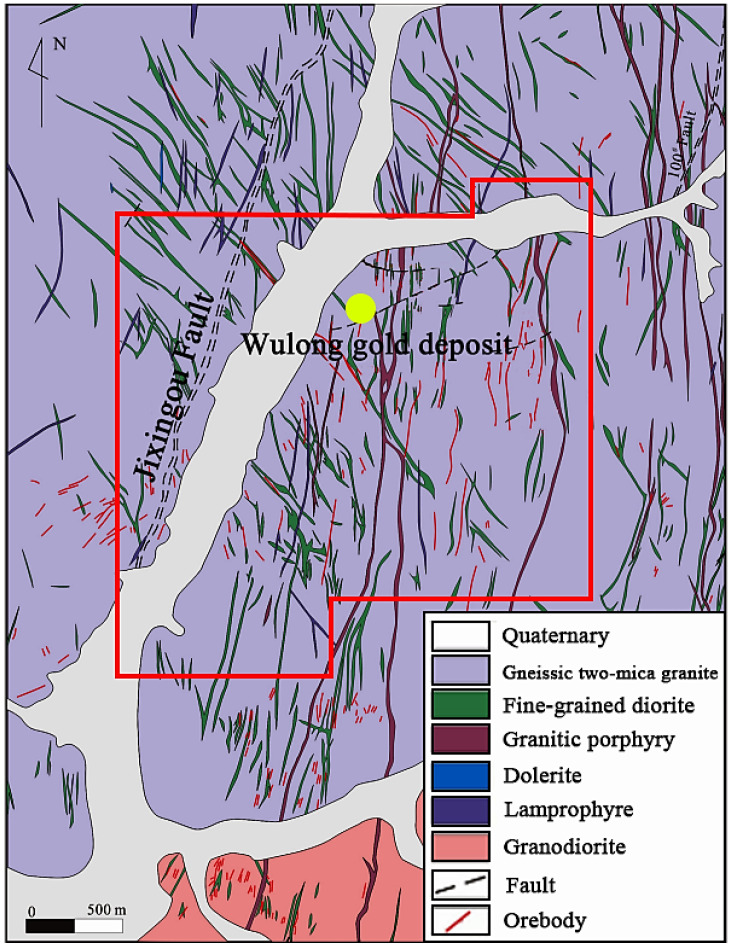
Geological map of the study area.

The Wulong gold deposit is located west of the Yalu River Fault, with a series of nearly parallel NNE-trending faults (Figure 1), including the Zhengjiapuzi Fault, Heigou Fault, Jixingou Fault, Yangjia Fault, Hongshi Fault, and Hanjiapu Fault, from east to west. These faults are several kilometers long and range in width from a few meters to 40 m, dipping 40–60° northwestward [29]. The Jixingou Fault (Figure 2) is near the Wulong gold deposit and is an important ore-bearing structure. The area within the red frame is the exploration area, covering the Wulong gold deposit. The Wulong gold deposit occurs in Late Jurassic gneissic two-mica granite and Early Cretaceous granodiorite. Early Cretaceous dikes are widely developed in the ore district, including pre-ore fine-grained diorite and granite porphyry, as well as post-ore thin-layered dolerite dikes.

The structure of this paper is as follows: Section 2 details the methods used in this study. Section 3 introduces the process of UAV compensation experiments and verifies the feasibility and advantages of the time–frequency joint Bi-LSTM in aeromagnetic compensation through two sets of experiments. Experiment a verifies the feasibility of aeromagnetic compensation combining time-domain and frequency-domain information by confirming the correlation between frequency characteristics and maneuvering actions. Experiment b demonstrates the improved compensation effect of Bi-LSTM integrating time–frequency information by comparing the compensation effects of different methods, and it conducts compensation experiments on actual aeromagnetic exploration data in the Dandong area to verify the method’s compensation effect in practical applications. Section 5 includes discussions on the method and experiments. Conclusions are summarized in Section 6.

## 2. Materials and Methods

**Figure 3:**
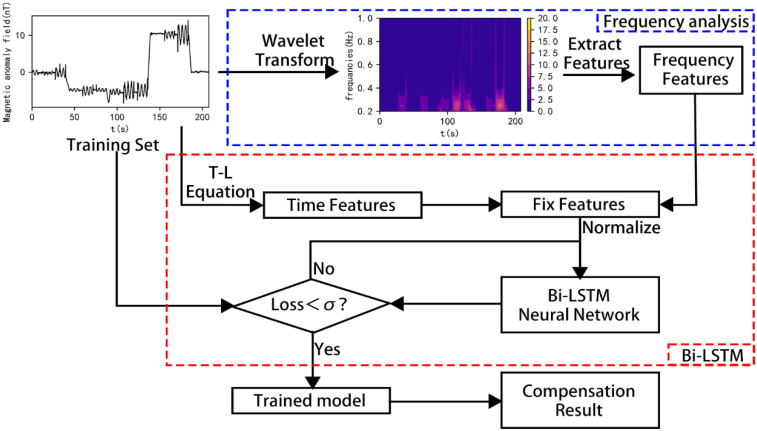
Flowchart of the aeromagnetic compensation method using the time–frequency combined Bi-LSTM network.

To remove the noise generated by the quadrotor UAV, a compensation flight is required before the actual flight to establish a compensation model for predicting the magnetic interference generated by the UAV during the actual flight. Wavelet analysis is used to extract the frequency characteristics contained in the UAV’s magnetic interference, analyze the frequency variation characteristics of the magnetic interference generated by the motor under various maneuvering actions of the UAV, and combine them with time characteristics. Bi-LSTM is used to learn the compensation model of the potential relationship between the magnetic interference generated by the UAV during various flights and the corresponding time characteristics and frequency characteristics, which is used to predict the magnetic interference generated by the quadrotor UAV during flight operations. The process of aeromagnetic compensation is shown in Figure 3.

### 2.1. Traditional Aeromagnetic Compensation Model

The magnetic interference generated by the UAV is closely related to its flight state. The T-L model proposed by Tolles and Lawson divides the interference signals generated by the UAV in aeromagnetic measurement into three parts according to the causes of generation, which are the constant field ($H_{p}$), the induced field ($H_{i}$), and the eddy current field ($H_{ec}$), which can be expressed as follows:

(1) $$H_{p}=\left|T\right|\left(c_{1}\cos\alpha +c_{2}\cos\beta +c_{3}\cos\gamma \right),$$

(2) $$H_{i}=\left|T\right|\left(\begin{array}{c} c_{4}\cos^{2}\alpha +c_{5}\cos\alpha \cos\beta +c_{6}\cos\alpha \cos\gamma +\\ c_{7}\cos^{2}\beta +c_{8}\cos\beta \cos\gamma +c_{9}\cos^{2}\gamma \end{array}\right),$$

(3) $$H_{ec}=\left|T\right|\left(\begin{array}{c} c_{10}\cos\alpha \;\cos^{\prime }\;\alpha +c_{11}\cos\beta \;\cos^{\prime }\;\alpha +c_{12}\cos\gamma \;\cos^{\prime }\;\alpha +\\ c_{13}\cos\alpha \;\cos^{\prime }\;\gamma +c_{14}\cos\beta \;\cos^{\prime }\;\gamma +c_{15}\cos\gamma \;\cos^{\prime }\;\gamma +\\ c_{16}\cos\alpha \;\cos^{\prime }\;\beta +c_{17}\cos\beta \;\cos^{\prime }\;\beta +c_{18}\cos\gamma \;\cos^{\prime }\;\beta \end{array}\right),$$

where T represents the Earth’s magnetic field; $\cos^{\prime }\;\alpha$, $\cos^{\prime }\;\beta$, and $\cos^{\prime }\;\gamma$ represent the derivatives of the direction cosines $\cos^{\prime }\;\alpha$, $\cos^{\prime }\;\beta$, and $\cos^{\prime }\;\gamma$ with respect to time t, respectively. Due to the inconsistent accuracy between the three-axis fluxgate magnetometer and the optically pumped magnetometer, errors will be generated that affect the calculation of direction cosines. Leach proposed a simplified direction cosine calculation method, which calculates direction cosines through fluxgate data:

(4) $$cos\alpha =\frac{T_{x}}{T_{t}}\;,cos\beta =\frac{T_{y}}{T_{t}}\;,cos\gamma =\frac{T_{z}}{T_{t}},$$

(5) $$\begin{array}{c} T_{t}=\sqrt{T_{x}^{2}+T_{y}^{2}+T_{z}^{2}}, \end{array}$$

$T_{x},{\;T}_{y}$, and $T_{z}$ are the three components of the fluxgate. The vector sum of the three components is used as the total magnetic field $T_{t}$ for calculating direction cosines. $H_{total}$ in the T-L equation is the total magnetic interference of the UAV measured by the optical pump magnetometer:

(6) $$H_{total}=H_{p}+H_{i}+H_{ec},$$

Combining Equations (1)–(3) and (6), the T-L equation can be simplified as follows:

(7) $$H_{total}=XS,$$

where X represents the direction cosines and their derivatives in Equations (1)–(3), which characterize the flight attitude of the UAV; S represents the 18 compensation coefficients from $c_{1}$ to $c_{18}$. By calculating these 18 compensation coefficients, a compensation model is established to determine the amplitude of magnetic interference signals generated by the UAV under different flight attitudes, thereby predicting UAV-generated magnetic interference in actual flights and removing it from mixed signals.

The traditional model solution method treats the T-L equation as a linear regression equation and solves it using the least squares (LS) method. The least squares solution of the T-L equation is as follows:

(8) $$S_{LS}={\left(X^{T}X\right)}^{-1}X^{T}H_{t},$$

Substitute the calculated compensation coefficient $S_{LS}$ back into Equation (7) to obtain the calculated value of magnetic interference ${\tilde{H}}_{LS}$. The formula for the compensation result ($H_{LS}$) is as follows:

(9) $$H_{LS}=H_{t}-{\tilde{H}}_{t}=H_{t}-XS_{LS},$$

In the calculation of the least squares method, there exists the term ${\left(X^{T}X\right)}^{-1}$. If there is perfect multicollinearity between independent variables, the rank of matrix *X* is less than (*N*+1) (where *N* is the number of independent variables), leading to $\left|X^{T}X\right|=0$ (${\left(X^{T}X\right)}^{-1}$ does not exist), making the LS method unsolvable. When $\left|X^{T}X\right|\approx 0$, the least squares parameter estimation will be inaccurate.

Leach proposed the ridge regression (RR) algorithm to address the multicollinearity problem in the T-L equation. By adding a regularization parameter k, it overcomes the possibility of near singularity when inverting $X^{T}X$ in the LS method, making the LS solution more stable. The RR coefficient S can be expressed as follows:

(10) $$S_{RR}={\left(X^{T}X+kI\right)}^{-1}X^{T}H_{t},$$

The calculated compensation coefficient $S_{RR}$ is substituted back into Equation (7) to obtain the calculated value of magnetic interference ${\tilde{H}}_{RR}$. The calculation formula for the compensation result ($H_{RR}$) is as follows:

(11) $$H_{RR}=H_{t}-{\tilde{H}}_{RR}=H_{t}-XS_{RR},$$

The classical T-L equation simply solves compensation coefficients, and the compensation accuracy depends on the solution accuracy of interference coefficients. To evaluate the performance of the compensation model, the standard deviation (STD) of magnetic interference before and after compensation and the Improvement Ratio (IR) can be calculated:

(12) $$STD=\sqrt{\frac{1}{n}\sum_{i=1}^{n}{\left({ℎ}_{i}-\bar{ℎ}\right)}^{2}},$$

(13) $$IR=\frac{{STD}_{u}}{{STD}_{c}},$$

In Equation (13), ${STD}_{u}$ is the standard deviation of raw aeromagnetic survey data; ${STD}_{c}$ is the standard deviation of data after aeromagnetic compensation. A smaller ${STD}_{c}$ indicates higher compensation accuracy, and a larger IR indicates better compensation effect.

### 2.2. Continuous Wavelet Transform

Callum Walter [27] analyzed the components of signals received during aeromagnetic exploration and found that the noise generated by the UAV is closely related to the rotation speed of the motors. In actual aeromagnetic detection, various noises are mixed in the time domain, affecting the time-domain data of the magnetometer. Simple filtering can remove high-frequency noise, but the low-frequency noise generated by the UAV overlaps with the low-frequency signals generated by underground targets in the frequency domain. Thus, it is impossible to effectively separate noise from target signals using only time-domain or frequency-domain analysis. This paper focuses on quadrotor UAVs and identifies and separates the UAV-generated magnetic interference by combining time-domain and frequency-domain analyses.

To combine frequency features with time-domain information as training sets for aeromagnetic compensation calculations, continuous wavelet transform is selected for time–frequency analysis in this paper. The formula for continuous wavelet transform is the following:

(14) $$W\left(\alpha ,\tau \right)=\frac{1}{\sqrt{\alpha }}\int_{-\infty }^{\infty }f\left(t\right)\psi^{*}\left(\frac{t-\tau }{\alpha }\right)dt,$$

where α is the scale parameter, controlling the stretching and shrinking of the basis function; different scale parameters correspond to wavelet bases of different frequencies; τ is the translation parameter, controlling the displacement of the basis function, corresponding to signals at different time segments; and $\psi \left(t\right)$ is the mother wavelet. In this paper, cgau8 is selected as the mother wavelet, and its formula is as follows:

(15) $$\psi \left(t\right)=(1-16t^{2}+\frac{256}{3}t^{4}-\frac{1024}{15}t^{6}+\frac{4096}{105}t^{8})\cdot e^{-4t^{2}}\cdot \left(-j2t\right),$$

Using cgau8 as the mother wavelet offers computational efficiency and local sensitivity. It avoids infinite interval integration when processing data within a limited interval, significantly reducing computational complexity, and it exhibits high sensitivity to local signal mutations, enabling accurate extraction of feature points. When applied in $L^{2}\left(R\right)$, substituting Equation (15) into Equation (14) yields the following:

(16) $$W_{c}\left(\alpha ,\tau \right)=\frac{1}{\sqrt{\alpha }}\int_{-\infty }^{\infty }x\left(t\right)\cdot \left[(1-16t^{2}+\frac{256}{3}t^{4}-\frac{1024}{15}t^{6}+\frac{4096}{105}t^{8})\cdot e^{-4{\frac{t-\tau }{\alpha }}^{2}}\cdot \left(j2\frac{t-\tau }{\alpha }\right)\right]dt,$$

**Figure 4:**
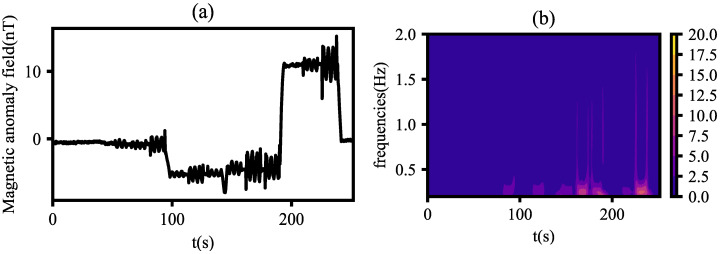
Waveform and time–frequency spectrum of magnetic interference in compensation flight: (**a**) original signal; (**b**) time–frequency spectrum.

In this paper, cgau8 is used for time–frequency analysis of signals, with the scale range set from 1 to 100, covering both large and small scales to ensure that the signal analysis includes both low-frequency and high-frequency features. Here, wavelet analysis is performed on magnetic interference from a compensation flight, and Figure 4a,b show the original signal and its corresponding time–frequency spectrum, respectively.

As can be seen from Figure 4, signal fluctuations in the original signal can be displayed in the time–frequency spectrum, indicating that continuous wavelet transform with cgau8 as the mother wavelet has good sensitivity to instantaneous signal fluctuations.

For the analysis of signals containing n time points, the time–frequency spectrum obtained after wavelet analysis can be regarded as a vector group $v=\left[v_{1},v_{2},\ldots ,v_{n}\right]$, where $v_{n}$ is a 1×i vector, i.e., $v_{n}=\left[v_{n1},v_{n2},\ldots ,v_{ni}\right]$, *i* represents the number of scales, and each value $v_{ni}$ in vector $v_{n}$ denotes the energy amplitude corresponding to a different frequency. If all frequency data in the time–frequency spectrum are used as training sets for neural network training, a large number of similar and useless low values will drown out effective information. This will instead reduce the learning speed and accuracy of the neural network. Therefore, it is necessary to further extract effective information from them.

(17) $$m_{i}=\mathrm{argmax}\left(v_{ni}\right),i\in \left\{1,2,\ldots ,100\right\},$$

(18) $$m=\left[m_{1},m_{2},\ldots ,m_{n}\right],$$

By converting the scales in matrix m into corresponding frequencies, the time–frequency feature matrix $F=\left[f_{1},f_{2},\ldots ,f_{n}\right]$ is obtained. In this paper, the frequency corresponding to the maximum amplitude at each sampling point is selected as the characteristic frequency of that sampling point. This characteristic frequency is used as the motor characteristic of the UAV to provide a basis for subsequent prediction of magnetic interference signals.

### 2.3. Bi-LSTM

The long short-term memory neural network (referred to as LSTM) is an improved Recurrent Neural Network (RNN) model. On the basis of RNN, LSTM networks prevent the problem of gradient vanishing or explosion caused by long-term dependence by adding screening and memory mechanisms to filter effective data, remove redundant data, and train the model. LSTM has excellent memory and generalization abilities for time-series data and can identify intrinsic connections in time-series data. LSTM is constructed based on three gate structures and cell states: the forget gate, input gate, and output gate. The mathematical principles of LSTM are as follows:

(19) $$f_{t}=\sigma \left(W_{f}\cdot \left[h_{t-1},x_{t}\right]+b_{f}\right),$$

(20) $$i_{t}=\sigma \left(W_{i}\cdot \left[h_{t-1},x_{t}\right]+b_{i}\right),$$

(21) $${\tilde{C}}_{t}=tanh\left(W_{C}\cdot \left[h_{t-1},x_{t}\right]+b_{C}\right),$$

(22) $$C_{t}=f_{t}\;*\;C_{t-1}+i_{t}\;*\;{\tilde{C}}_{t},$$

(23) $$o_{t}=\sigma \left(W_{o}\cdot \left[h_{t-1},x_{t}\right]+b_{o}\right),$$

(24) $$h_{t}=o_{t}\;*\;\tanh\left(C_{t}\right),$$

where $W_{f}$ is the weight matrix, $b_{f}$ is the bias vector, $\left[h_{t-1},x_{t}\right]$ represents the concatenated vector of the previous hidden state $h_{t-1}$ and the current input $x_{t}$, and ${\tilde{C}}_{t}$ is the candidate cell state for updating information. At the input gate, ${\tilde{C}}_{t}$ and historical cell state $C_{t-1}$ are selectively retained, and the cell state $C_{t}$ is updated in real-time. The hidden state $h_{t}$ at the current moment is obtained by fusing the output gate $o_{t}$ with the result of the nonlinear transformation of the cell state $C_{t}$, containing both current sequence features and long-term dependence information.

Chen [21] detailed the principle and process of Bi-LSTM and proved that Bi-LSTM can effectively capture the data correlation between time series obtained by fluxgate magnetometers, showing great potential in magnetic anomaly signal processing.

Before inputting data into the Bi-LSTM network for training, it is necessary to concatenate the time-domain information and frequency-domain information. Let the time-domain information be a matrix of length t, with 18 attitude parameters at each time point; then, the time-domain information can be expressed as $X_{18,t}$:

(25) $$X_{18,t}=\left[\begin{array}{ccc} \begin{array}{cc} \gamma_{1,1} & \gamma_{1,2}\\ \gamma_{2,1} & \gamma_{2,2} \end{array} & \ldots & \begin{array}{c} \gamma_{1,1}\\ \gamma_{2,t} \end{array}\\ \vdots & \ddots & \vdots \\ \begin{array}{cc} \gamma_{18,1} & \gamma_{18,2} \end{array} & \ldots & \gamma_{18,t} \end{array}\right],$$

The above 18 parameters represent the UAV’s attitude. Wavelet analysis is used to extract the motor frequency characteristics corresponding to the UAV under different maneuvering attitudes from the magnetometer signals. The characteristic frequencies of the motors and the attitude parameters are concatenated in chronological order to obtain the input matrix:

(26) $$\begin{array}{c} x_{19,t}=\left[\begin{array}{ccc} \begin{array}{cc} \gamma_{1,1} & \gamma_{1,2}\\ \gamma_{2,1} & \gamma_{2,2} \end{array} & \ldots & \begin{array}{c} \gamma_{1,1}\\ \gamma_{2,t} \end{array}\\ \vdots & \ddots & \vdots \\ \begin{array}{cc} \begin{array}{c} \gamma_{18,1}\\ f_{1} \end{array} & \begin{array}{c} \gamma_{18,2}\\ f_{2} \end{array} \end{array} & \ldots & \begin{array}{c} \gamma_{18,t}\\ f_{t} \end{array} \end{array}\right]\\ =\left[\begin{array}{ccc} x_{1} & x_{2} & \begin{array}{cc} \ldots & x_{t} \end{array} \end{array}\right] \end{array},$$

**Figure 5:**
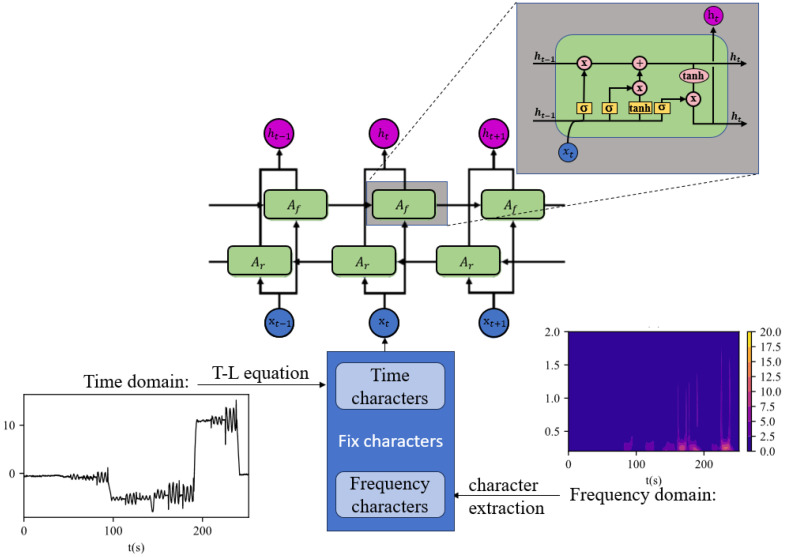
Schematic diagram of the compensation network structure.

$x_{19,t}$ is a mixed feature matrix containing both UAV attitude information and motor frequency information. The actual network training process is illustrated in Figure 5. 

Input $x_{19,t}$ into the Bi-LSTM network to predict the magnetic interference generated by the quadrotor UAV, with the goal of minimizing the Mean Squared Error (MSE) between the predicted magnetic interference and the actual magnetic interference:

(27) $$Loss\left(\theta \right)=\frac{1}{N}\sum_{t=1}^{N}{\left(T_{t}-h_{t}\right)}^{2},$$

where $T_{t}$ is the actual magnetic interference of the UAV, and $h_{t}$ is the magnetic interference predicted by the network. In this paper, the Adam optimizer is used to optimize the Bi-LSTM network. The initial learning rate is set to 0.001, and the learning rate is adaptively adjusted while the network learns data correlations. The training process is terminated early when the Loss is less than 0.0001. This optimization improves the robustness and learning speed of the network.

## 3. Results

To verify the feasibility of extracting frequency features of quadcopter UAV magnetic interference from compensation flight detection signals and applying them to aeromagnetic compensation model estimation, and to prove that combining time-domain and frequency features can improve aeromagnetic compensation accuracy, two sets of experiments are conducted in this paper. Experiment a is designed to verify the feasibility of using frequency features for UAV aeromagnetic compensation model estimation. Experiment b demonstrates that Bi-LSTM can improve aeromagnetic compensation accuracy, and Bi-LSTM combining time–frequency information extracted by wavelet transform with time-domain information can further enhance the compensation effect.

### 3.1. UAV Compensation Flight

**Figure 6:**
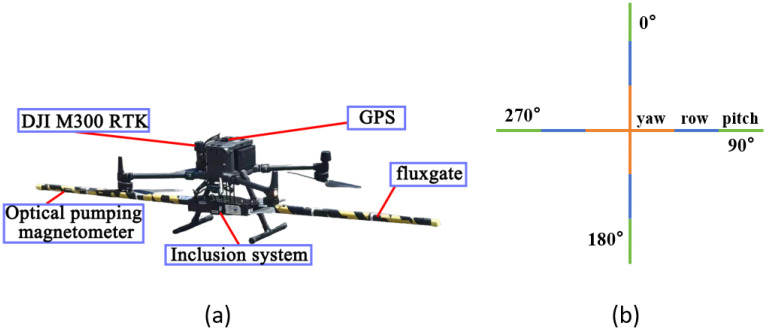
Experimental instruments and flight route: (**a**) experimental UAV and instruments; (**b**) compensation flight route.

The experiment uses a DJI M300 RTK as the flight platform, a quadcopter UAV equipped with a high-precision optical pump magnetometer and a supporting triaxial fluxgate magnetometer. The two magnetic probes are installed at the ends of carbon fiber rods extending from the left and right sides of the UAV’s landing gear, measuring changes in the total magnetic field strength and three mutually orthogonal magnetic field components (Figure 6a).

To obtain magnetic interference generated by the UAV during various maneuvering actions for aeromagnetic compensation, compensation flights need to be conducted before actual flights. The compensation flight involves three sets of maneuvering flights: yaw (±5°), roll (±10°), and pitch (±5°) in four directions. Compared with fixed-wing aircraft, multi-rotor UAVs have hovering capabilities and more flexible steering, enabling steering in a smaller space during actual operations. Thus, compensation flights can be completed in a small space, ensuring that ground magnetometers can fully collect magnetic interference generated by the UAV during compensation flights. The top view of the UAV’s compensation flight route in this paper is shown in Figure 6b.

### 3.2. Experiment a: Verification of Correlation Between Frequency Features and Maneuvering Actions

**Figure 7:**
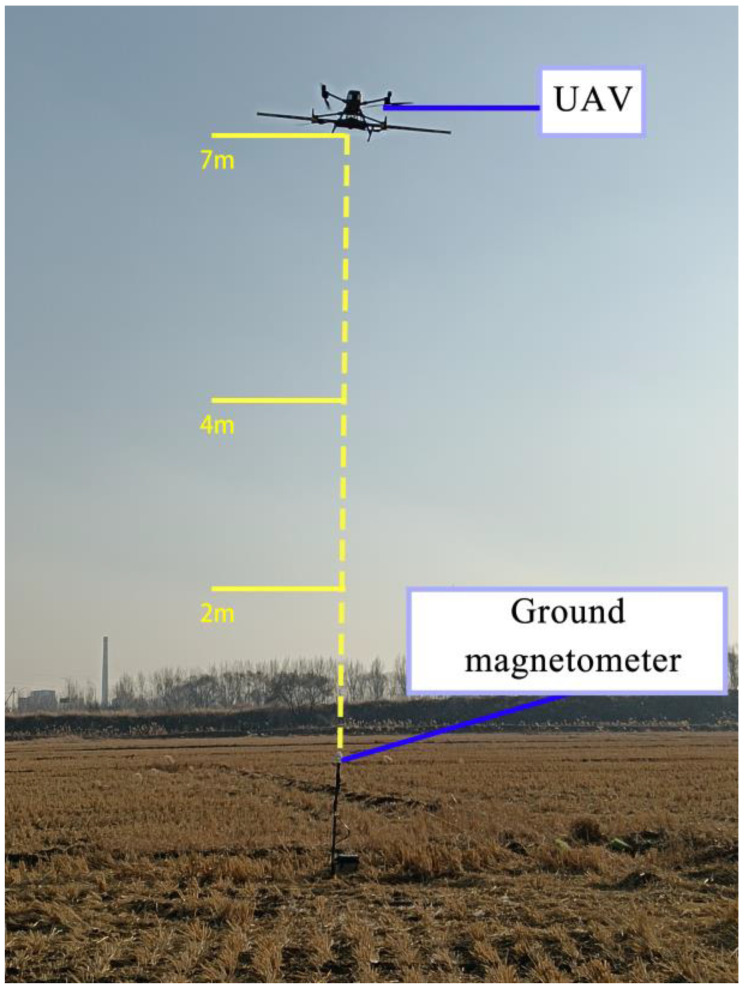
Scene diagram of compensation flight experiment.

Fourier transform reveals similar frequency components in compensation flight signals, but it loses time information and cannot be used for magnetic interference estimation. Time–frequency spectra obtained by wavelet transform of signals show frequency changes over time, requiring verification of the strong correlation between frequency changes and maneuvering actions. Three flight experiments were conducted (Figure 7), with the UAV performing compensation flights at heights of 2 m, 4 m, and 7 m above the ground. Each compensation flight followed the same sequence of actions and duration, with a total duration of 200 s.

**Figure 8:**
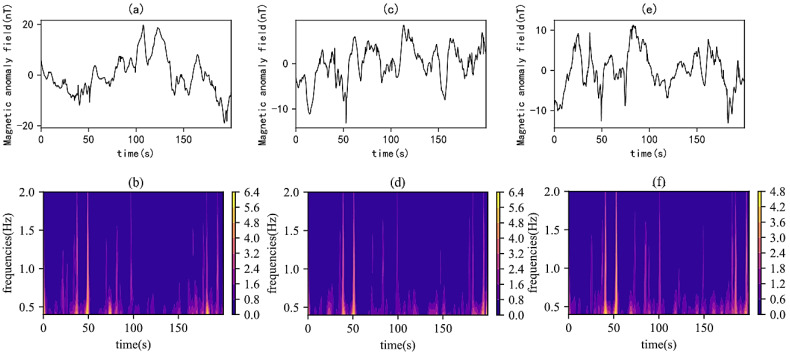
Waveforms and time–frequency spectra of magnetometer signals from the flight experiments: (**a**) original signal of the first experiment; (**b**) time–frequency spectrum of the first experiment; (**c**) original signal of the second experiment; (**d**) time–frequency spectrum of the second experiment; (**e**) original signal of the third experiment; (**f**) time–frequency spectrum of the third experiment.

A ground magnetometer was placed below the UAV to collect magnetic interference generated by the UAV during compensation flights. The UAV performed the same compensation flights at 2 m, 4 m, and 7 m above the magnetometer, and wavelet analysis was conducted on the signals collected by the magnetometer. Figure 8 shows the original signal waveforms and corresponding time–frequency spectra obtained from flight experiments at various heights.

In the experiments, by observing the original signals and time–frequency spectra of multiple experiments (Figure 8), it was found that frequency amplitude variations occurred in the same time segments of the time–frequency spectra. As the distance between the UAV and the magnetometer increased, the signal intensity decreased, and the amplitude in the time–frequency spectrum also decreased gradually. Although the frequency amplitudes were different, the frequency variation patterns showed similarities. This confirms that the UAV generates the same frequency characteristic variations when performing the same maneuvering actions, indicating a strong correlation between the UAV’s maneuvering actions and frequency characteristics.

### 3.3. Experiment b: Bi-LSTM Network Compensation Method Combining Time and Frequency

Experiment a proves a strong correlation between UAV maneuvering actions and magnetic interference frequencies, and compensation parameters in aeromagnetic compensation calculations are also strongly correlated with UAV maneuvering actions. Therefore, magnetic interference frequency features are used as constraint terms in the aeromagnetic compensation process, and time-domain and frequency-domain information are combined and input into the Bi-LSTM network for model training to extract potential correlations, resulting in an optimized compensation model, referred to as Bi-FT (Time–Frequency Joint Bi-LSTM) in this paper.

**Figure 9:**
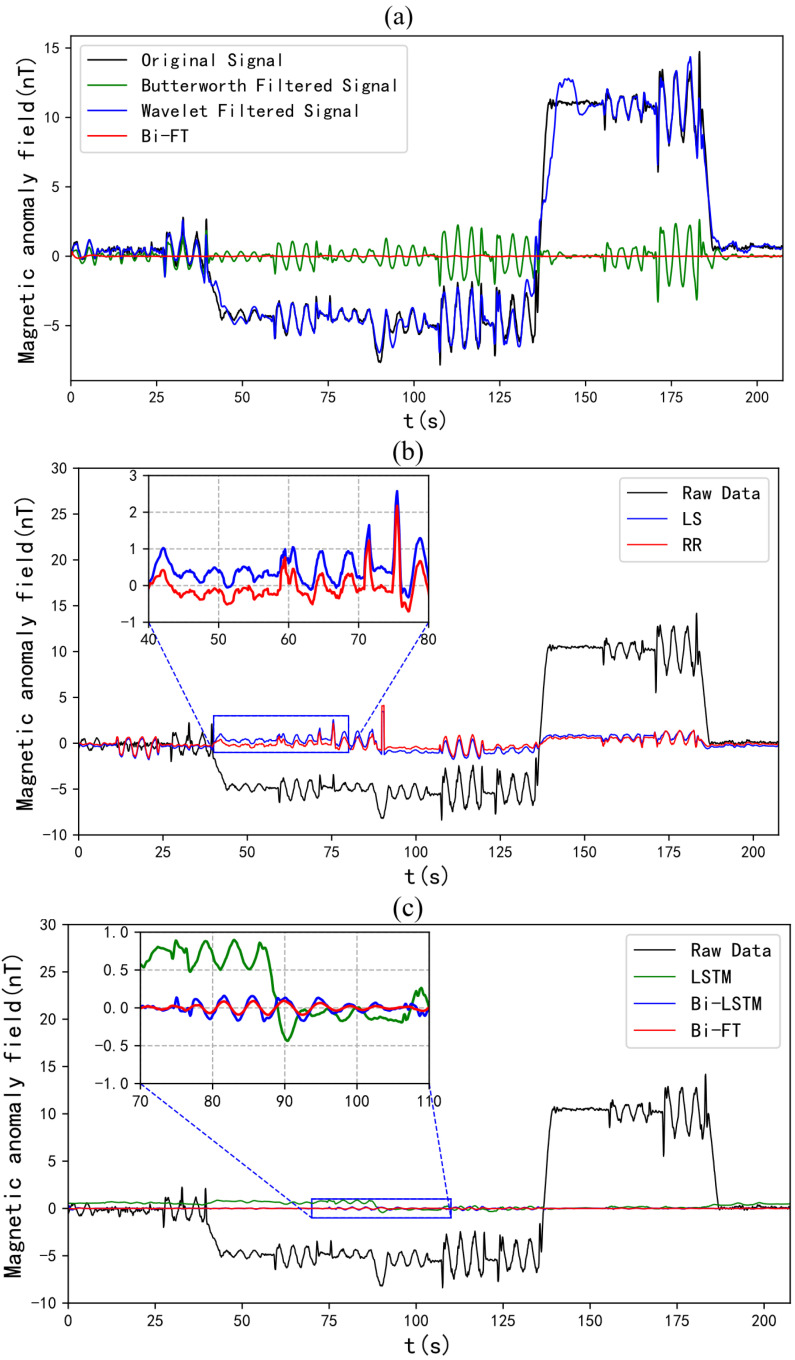
Compensation effect diagrams of various methods: (**a**) Butterworth filter, wavelet filter, and Bi-FT; (**b**) least square and ridge regression; (**c**) LSTM, Bi-LSTM, and Bi-FT.

When only filters were used for noise removal, the Butterworth filter achieved significantly better denoising results than the wavelet filter. This is because Fourier transform treats signal jumps as high-frequency signals and removes them, while wavelet analysis often treats them as low-frequency signals and retains them. Compared with traditional linear compensation methods and LSTM, Bi-LSTM had a higher IR. The IR of the compensation results of Bi-FT, which integrates time–frequency information, was further higher than that of Bi-LSTM or LSTM alone, and it had the highest IR among all of the aforementioned methods. Combining the compensation results (Figure 9, Table 1), it can be concluded that Bi-FT achieved the best compensation effect compared with the other methods mentioned above.

Finally, an additional test experiment was conducted on the Bi-FT method to evaluate its stability and robustness. In the test experiment, the same compensation method was used, and the compensation models trained with Dataset 1 and Dataset 2 were used to compensate Dataset 2 and Dataset 1, respectively. The Cross-Calibration Index (CCI) [30,31] between the two compensation results was calculated. The experimental results showed that the CCI was 1.02, which is very close to 1, indicating that the Bi-LSTM compensation method has excellent robustness and stability.

## 4. Line Flight Test

**Figure 10:**
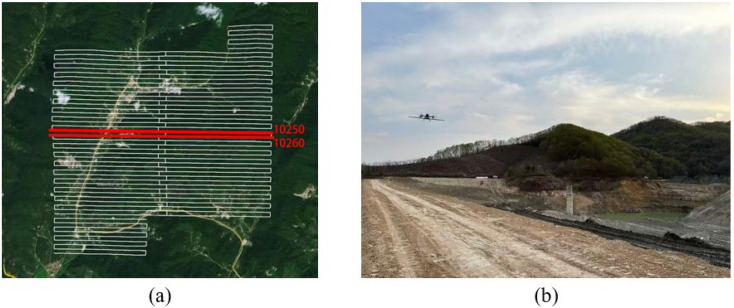
(**a**) satellite remote sensing image of the detection area and locations of Lines 10250 and 10260. (**b**) photos of the actual exploration flight operations.

To further verify the compensation effect of the method, the Bi-FT compensation method was applied to actual flights. In this study, a DJI quadrotor UAV (DJI Technology Co., Ltd., Shenzhen, China) was used to carry an airborne magnetic survey system (GTK Technology (Shenzhen) Co., Ltd., Shenzhen, China) consisting of an optical pump magnetometer and a triaxial fluxgate magnetometer for field flight operations. According to existing geological data, there are NNE-, NW-, and NS-trending structures in the study area [29] The survey area has a relatively regular shape, covering an area of approximately 20 km^2^. A total of 52 survey lines were arranged, with an east–west direction and a line spacing of 100 m. The UAV flight height was 100 m. Figure 10a shows the satellite remote sensing image of the detection area and the flight path. A terrain-following flight mode was adopted to maintain the UAV’s height above the ground at approximately 100 m during the flight survey. Figure 10b shows the actual flight operation.

**Figure 11:**
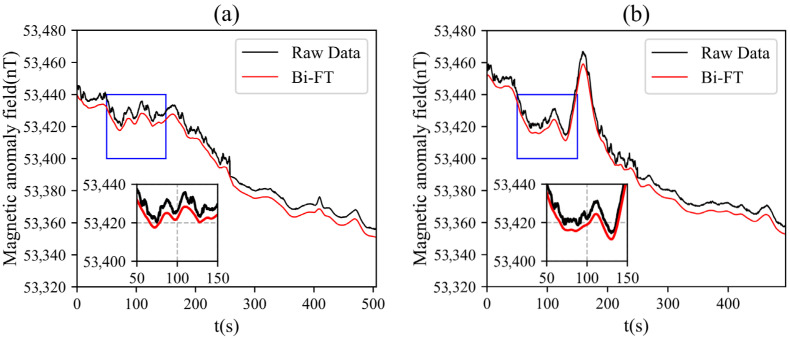
Magnetic anomaly profiles of survey lines: (**a**) original signal and compensation result of Line 10250; (**b**) original signal and compensation result of Line 10260.

Taking Line 10250 (northbound) and Line 10260 (southbound) as examples, the original magnetic anomalies and the magnetic anomaly profiles after compensation using the Bi-FT method are shown (Figure 11). As observed in Figure 11, the original signals contained a large number of serrated edges and irregular anomaly interferences, some of which were caused by maneuvering noise during UAV detection. This affects the resolution of the magnetic anomaly map and reduces the accuracy of subsequent inversion work. After compensating the original data using Bi-FT, the interference generated by the UAV was removed, and the data became smoother, providing better data for subsequent inversion and interpretation work.

**Figure 12:**
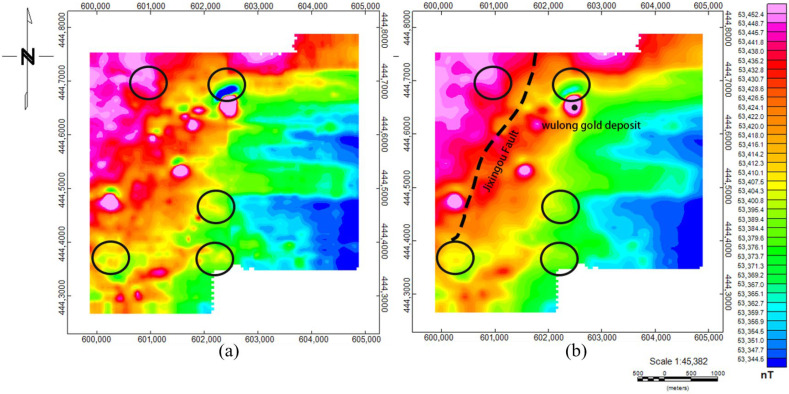
Schematic diagrams of magnetic anomalies in the study area: (**a**) original magnetic anomalies; (**b**) magnetic anomalies after compensation.

The compensation method was applied to all survey line data. Figure 12a shows the magnetic anomaly map of the original data (diurnal variation effects were eliminated before aeromagnetic compensation). The pre-compensation magnetic anomalies contain maneuver noise generated during aeromagnetic exploration, which affects the resolution of the magnetic anomaly map and severely impacts subsequent inversion and interpretation. Figure 12b shows the magnetic anomaly results after compensation using the Bi-FT method. Comparing the magnetic anomalies within the black frame, high-frequency anomalous signals generated by the UAV were eliminated, making the shape and location of low-frequency anomalies more distinct. Moreover, the magnetic anomalies are consistent with the strike of the known Jixingou Fault in the study area, and corresponding high magnetic anomaly values exist at the Wulong gold deposit.

## 5. Discussion

Aeromagnetic exploration model solution methods based on linear regression have been widely studied, but there are few studies on the impact of UAV heading and attitude changes on the frequency characteristics of magnetic interference. This study confirmed through experiments that when a quadrotor UAV performs the same maneuver, the time–frequency spectrum of its magnetic interference shows a consistent frequency variation pattern—this finding provides a physical basis for introducing time–frequency characteristics into the aeromagnetic compensation model. Experimental results show that the Bi-LSTM compensation method (Bi-FT) based on time–frequency joint representation in quadrotor UAV aeromagnetic exploration is significantly superior to frequency-domain-only filtering methods (Butterworth filter, wavelet filter), traditional linear methods (least squares method, ridge regression), and neural network methods without fused time–frequency characteristics (LSTM, Bi-LSTM) in terms of compensation accuracy and robustness—especially in the Improvement Ratio (IR) index, verifying the effectiveness of time–frequency information fusion in improving compensation performance.

However, this study has certain limitations. Firstly, the applicability of the method to other types of UAVs (such as hexacopters, fixed-wing UAVs) has not been verified. Different types of UAVs have differences in power systems and structural designs, and their magnetic interference frequency features may differ from those of quadcopter UAVs, so the generalizability of the Bi-FT method requires more experimental verification. Additionally, in actual survey experiments, although the UAV maintained a height of 100 m relative to the ground, terrain undulations in the survey area may cause changes in altitude, and whether the resulting magnetic gradient effect affects compensation accuracy has not been verified. Therefore, future research may consider introducing height parameters as model inputs to construct a spatiotemporal joint compensation model, adapting to aeromagnetic detection needs under complex terrain conditions.

Meanwhile, the computational complexity of the Bi-FT method in this study is slightly higher than that of traditional linear methods, mainly due to the time–frequency feature extraction via continuous wavelet transform and the training process of the Bi-LSTM network. In practical engineering applications, optimizing computational efficiency while ensuring compensation accuracy and shortening real-time compensation response time is a key issue to be addressed in subsequent work.

## 6. Conclusions

Quadcopter UAVs have been widely used in aeromagnetic surveying due to their advantages of low cost, wide coverage, and high operational efficiency. However, magnetic interference generated by the platform itself significantly reduces the quality of detection data, and aeromagnetic compensation is a key step toward improving data effectiveness. To address the problem that traditional methods struggle to handle time–frequency aliased noise, this study proposes a time–frequency joint representation neural network-based aeromagnetic compensation method. Continuous wavelet transform (CWT) is used to extract time–frequency features of magnetic interference, which are combined with raw time-domain data and input into a bidirectional long short-term memory network (Bi-LSTM) to achieve accurate prediction and compensation of UAV magnetic interference.

Experimental verification shows the following:

When performing the same maneuver, quadrotor UAVs show a consistent frequency variation pattern in the time–frequency spectrum of magnetic interference, confirming that frequency characteristics can be used as effective constraint terms for aeromagnetic compensation.

After Bi-FT compensation, the residual interference magnetic field is less than 0.5nT; its standard deviation (STD) is lower than that of filtering methods (Butterworth filter, wavelet filter), traditional linear methods (least squares method, ridge regression), and neural network methods without fused time–frequency characteristics (LSTM, Bi-LSTM), with a maximum Improvement Ratio (IR) of 22.123 and a Cross-Calibration Index (CCI) close to 1—indicating excellent compensation accuracy and robustness.

In actual aeromagnetic exploration in Dandong, the Bi-FT method effectively removes UAV maneuvering noise, making magnetic anomalies more consistent with the positions and boundaries of known structures, and providing a high-quality data foundation for subsequent inversion and geological interpretation. The time–frequency joint compensation method proposed in this study provides a new solution for magnetic interference suppression in quadcopter UAV aeromagnetic surveying, and it has important application value, especially in fields with high data accuracy requirements such as mineral exploration.

## Figures and Tables

**Table 1:** Comparison of compensation effects of various methods.

Methods	${STD}_{u}$	${STD}_{c}$	IR
Butterworth filter	6.059	0.729	8.311
Wavelet filter		6.002	1.009
Least Square		1.042	5.813
Ridge Regression		0.947	6.398
LSTM		0.358	15.557
Bi-LSTM		0.296	19.229
Bi-FT		0.257	22.123

## Data Availability

The data presented in this study are available on request from the corresponding author. The data are not publicly available because they have been provided by a classified project.
